# Aging Compensation in a Class-A High-Frequency Amplifier with DC Temperature Measurements

**DOI:** 10.3390/s23167069

**Published:** 2023-08-10

**Authors:** Josep Altet, Xavier Aragones, Enrique Barajas, Xavier Gisbert, Sergio Martínez, Diego Mateo

**Affiliations:** Electronic Engineering Department, Universitat Politcnica de Catalunya-Barcelona Tech, 08034 Barcelona, Spain; xavier.aragones@upc.edu (X.A.);

**Keywords:** CMOS integrated circuits, aging, aging monitoring, aging compensation, temperature measurements, differential temperature sensors

## Abstract

One of the threats to nanometric CMOS analog circuit reliability is circuit performance degradation due to transistor aging. To extend circuit operating life, the bias of the main devices within the circuit must be adjusted while the aging degradation process affects them by using a monitor circuit that tracks the evolution of the circuit performance. In this paper, we propose the use of DC temperature measurements in the proximity of the circuit to perform the monitoring of circuit performance degradation and as an observable variable to adjust the bias of the main devices to restore the degraded performance to the original values. To this end, we present experimental results obtained from nine samples of a standard CMOS integrated circuit containing a high-frequency class-A power amplifier and a differential temperature sensor. After accelerated aging, the gain of the amplifier is degraded up to 50%. We propose two different procedures to perform DC temperature measurements that allow tracking of the amplifier gain degradation due to aging and, by uniquely observing temperature readings, automatically set a new bias for the amplifier devices that restores the original amplifier gain. Whereas one of the procedures is able to restore the gain up to a certain limit, the second allows full gain restoration.

## 1. Introduction

One of the well-known consequences of the scaling of Complementary Metal-Oxide-Semiconductor (CMOS) microelectronic technologies is the increasing importance of variability issues [1]. A basic classification distinguishes between zero-time variability associated with IC manufacturing (inter- or intra-die process variability) and variations that appear along the operation lifetime of the circuit. Among the second ones, the temperature at which the circuit works may change along time because of external (ambient) or internal (power dissipation) reasons, and this affects the performance of sensitive circuits in the IC. Supply voltage $V_{DD}$ can also suffer variations, for instance due to DC (IR drop) or in the form of AC ripple. Process Design Kits (PDKs) of commercial CMOS technologies include device models to account for the effects of Process, $V_{DD}$ and Temperature (PVT) variations, and thus these are commonly accounted for during the design of the integrated circuit. This means that techniques are commonly applied to make the circuits robust against the expected effects of PVT variability. Nevertheless, voltage and temperature are not the only sources of time variability. In the last decade, the effects of aging have become an increasing source of concern for IC designers.

In CMOS technologies, aging is the result of different physical phenomena: Hot Carrier Injection (HCI), Bias Temperature Instability (BTI), Time-Dependent Dielectric Breakdown (TDDB) and Electro-Migration (EM). Major reliability concerns are related to TDDB and EM effects because of their potential to produce catastrophic failures. On the contrary, HCI and BTI produce a progressive circuit wearout and have attracted increasing interest [2]. HCI was first observed in old 2 μm technologies [3] and originates from high-energy carriers that become trapped in the oxide or create interface states and is thus associated with the existence of high lateral electric fields. BTI phenomena also create oxide and interface traps, but in this case it is because of the vertical electric fields in the oxide. MOS operating conditions thus determine the dominance of HCI or BTI phenomena. The reported consequence of both HCI and BTI effects is an increase in the device threshold voltage $V_{TH}$ and a decrease in the saturation current $I_{DS}$ [4,5].

The effects of HCI and BTI aging phenomena increase both with time and with the electrical fields [2]. This is used in experimental studies to accelerate aging phenomena by operating the circuit above nominal voltages, thus stressing the devices. This allows us to observe, after a reasonable time, aging degradation that otherwise would take years to show [6,7,8]. Also, this means that aging degradation is naturally more important in devices that operate under large electrical fields. This is the case of Power Amplifiers (PA) in line drivers or RF transmitters, which are circuits in which aging produces degradation of gain, saturated output power, decreased power efficiency or even linearity [8,9,10,11,12].

The consequences of MOSFET aging in a given circuit can be accounted for using specific model cards and simulators; then, during the design process, the circuit can be overdesigned anticipating a worst-case scenario [13]. An alternative and often more economical solution to obtain robustness against the effects of aging is the knobs-and-monitors approach [14,15]: add some circuitry that senses and monitors the circuit degradation and use this information to actuate some control knob in the aged circuit. In the context of digital circuits, the classical approach to monitor aging degradation is to detect delay increase by means of ring oscillator circuits, which are sometimes configured as odometers [16,17,18]. The information of these aging sensors can then be used to increase $V_{DD}$ dynamically, thus recovering performance [18]. In the case of amplifiers, while body biasing has been proposed as a control knob in [19], acting on the DC biasing is a more convenient mechanism to correct gain degradation. In the specific case of PA circuits, a common approach to sense their performance is to add a power sensor connected to the output. This has been used in [20,21] to monitor the power of the transmitted signal with the objective of dynamically optimizing the trade-off between power efficiency and linearity, which is achieved by tuning the transistor biasing and thus its conduction angle. In [22], the output power is monitored to fix load impedance mismatches produced along time, which result in degraded signal power and efficiency. In none of these cases were the sensors demonstrated to monitor aging wearout.

Using a power sensor connected to the PA output is an inconvenient solution since it adds electrical loading to the output node, thus producing impedance mismatch and forcing a PA-sensor co-design. A noninvasive alternative to power sensors is to monitor the amplifier’s response with temperature measurements with the purpose of detecting variations in the power dissipated by the MOS transistors. Such temperature measurements can be done using Infra-Red (IR) imaging techniques [23], but it is most convenient to embed a differential sensor circuit integrated in the same chip and close to the PA being monitored. This has been proposed in [22,24] to monitor and optimize the efficiency in PA circuits, in [25] to monitor output mismatches and in [26] to monitor the output power and gain of class-A linear amplifiers. In all these cases, the PA performance variation was forced either by tuning its DC biasing or with a tunable load: never after real aging degradation of MOS transistors. On the other hand, while a differential sensor circuit can provide high-temperature sensitivity, it becomes highly affected by process variations and mismatches. This complicates its usage in producing automatic self-healing correction, which actually is done only manually in most of the literature [24,27]. In [22], automatic self-calibration is demonstrated, but the authors use a complex algorithm that combines the information of temperature sensors with that of DC and power sensors.

**Figure 1:**
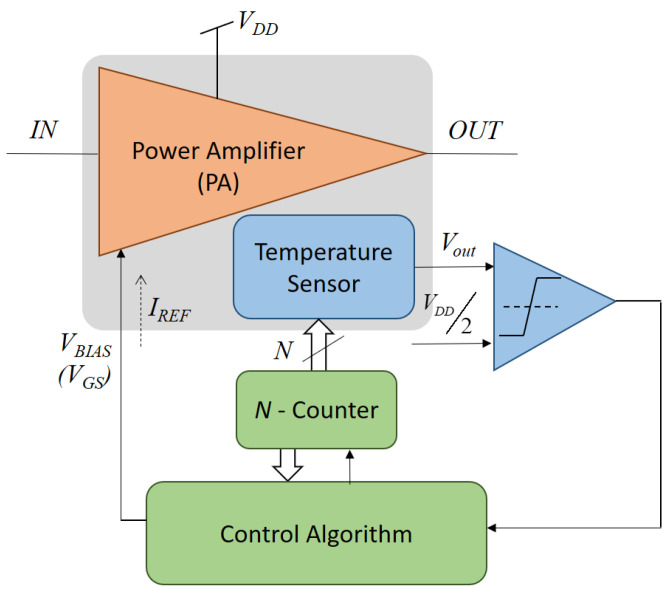
Simplified block diagram of the self-calibration solution to monitor and compensate for the effects of aging on a power amplifier based on the DC reading of a temperature sensor.

In this paper, we validate experimentally how the DC reading of a differential temperature sensor can be used to monitor the effects of aging in a CMOS PA circuit and how this reading can be used in a self-healing loop to preserve the PA nominal performance along time. Compared to previous solutions based on power sensors, the proposed approach does not require any circuit redesign and can be added to any existing PA circuit simply by placing a sensitive device next to a PA transistor dissipating power, while the rest of the sensor circuit can be placed elsewhere. Figure 1 shows a simplified scheme of the implemented solution. While the general scheme fits a classical knobs-and-monitor approach, the distinctive contributions of this work are (i) two different self-healing procedures that use the DC reading of a differential temperature sensor are evaluated, showing an interesting trade-off between simplicity and accuracy; (ii) the solution is validated experimentally after producing real (accelerated) aging on the PA circuit; (iii) the effects of process variability on the thermal sensor as well as on the PA circuit are addressed by the algorithms proposed, and (iv) automatic self-healing is demonstrated experimentally. This experimental validation is performed on a test IC that contains a linear PA circuit together with the differential temperature sensor (greyed region in Figure 1). While the rest of the blocs in Figure 1 are off-chip in our setup, they would benefit from the scaling down of CMOS technologies and thus can be added on-chip at a very reduced cost.

The paper is organized as follows: Section 2 presents a brief description of the integrated circuit together with the experimental characterization of the PA circuit, the differential temperature sensor and the thermal coupling between the PA and the temperature sensor. Throughout this paper, we use two different measurement procedures, whose principles and descriptions are explained in Section 3. There, we validate these techniques by reporting how DC temperature sensors can track the gain variations produced after tuning the bias of the PA. Section 4 reports the accelerated aging procedure performed on the PA. It presents how the PA’s figures of merit degrade because of aging and how to monitor the gain degradation along time by using only temperature measurements. The section also shows how to keep the gain constant by using only those temperature readings and actuating the PA’s DC bias. Finally, Section 5 concludes the paper.

## 2. Experimental Setup

### 2.1. Circuit Description

In order to demonstrate the use of temperature sensors to monitor and compensate for the aging degradation in amplifiers, a test Integrated Circuit (IC) has been designed on low-cost 0.35 µm CMOS technology containing a linear PA circuit together with a differential temperature sensor (Figure 1). While the design and simulations of the PA circuit and sensor have already been described in [28], a brief summary of their characteristics is provided in this section together with their experimental characterization.

**Figure 2:**
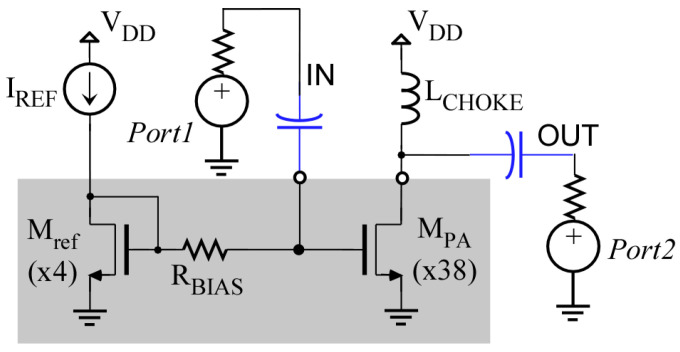
Scheme of the Power Amplifier circuit used as a CUT. The region greyed out indicates components integrated in the IC. Numbers (×4) and (×38) indicate the number of unit transistors.

The Circuit Under Test (CUT) is a single-stage class-A wideband power amplifier (PA) consisting of a common-source transistor loaded with an off-chip choke inductor (Figure 2). External networks (not shown in the figure) provide input and output impedance-matching to 50 Ω in the frequency range of operation. While the reference transistor in the current mirror would be driven with a constant current $I_{REF}$, in our experiments, we used a voltage source, thus directly controlling the gate-to-source $V_{GS}$ voltage of both transistors.

**Figure 3:**
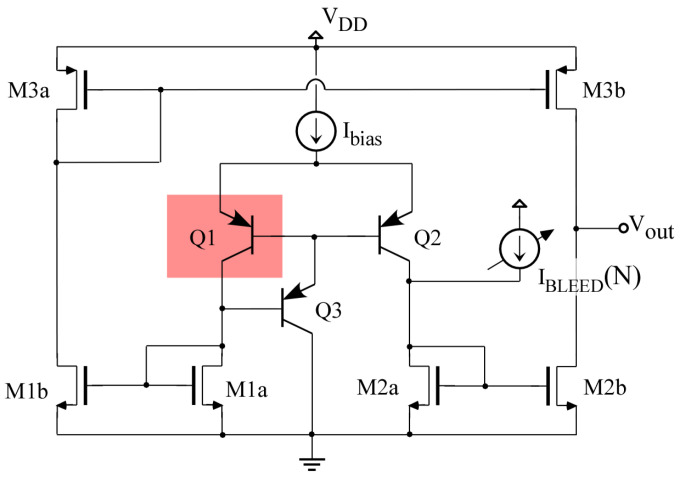
Schematic of the temperature sensor with current bleeding for extended dynamic range and variability compensation.

**Figure 4:**
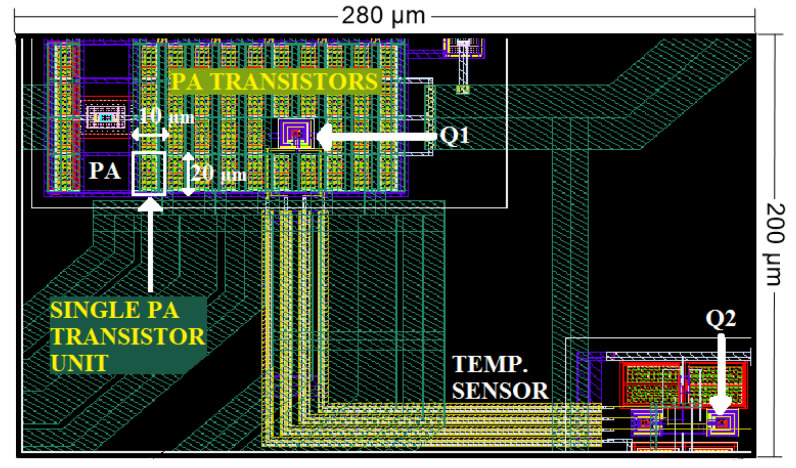
Detail of one of the CUTs and a temperature sensor, highlighting the location of temperature transducers Q1 and Q2.

Together with the amplifier, a differential temperature sensor of high sensitivity and dynamic range, described in detail in [28], has been implemented. As shown in Figure 3, it consists of an Operational Transconductance Amplifier (OTA) circuit whose differential input pair is imbalanced due to the temperature difference between bipolar transistors *Q1* and *Q2*, which act as temperature transducers. By placing transducer *Q1* together with the CUT and *Q2* away from it, a temperature imbalance between both temperature transducers is produced as a consequence of the power dissipated by the CUT, which is the magnitude to be sensed. Detailed analysis and characterization of differential temperature sensors implemented in the same technology are available in [29]. Figure 4 shows a detail of the IC layout, showing the placement of the PA transistors together with the sensor circuit and detailing the position of the temperature transducers.

While the high output resistance of the OTA circuit provides high sensitivity and thus fine temperature resolution (e.g., [29] reports a sensitivity of 7.5 V/C), it also produces a highly limited linear dynamic range. Also, manufacturing process variability and mismatches will easily produce a saturated output. In order to extend the dynamic range as well as compensate for the effects of manufacturing variability, similarly to [29], a bleeding current $I_{BLEED}\left(N\right)$ is added to one of the transducer currents, as shown in Figure 3. This bleeding current is controlled with a 10-bit digital input *N*, sized according to the targeted dynamic range and resolution [28]. The variations in the sensor output voltage when within the linear range can be written as:

(1) $$\Delta V_{OUT}=Sens\cdot \Delta T+K\cdot R_{OUT}\cdot I_{BLEED}\left[N\right]$$

(2) $$\Delta T=R_{TH}\cdot P_{PA}$$

where *Sens* is the sensitivity of the sensor circuit $\partial V_{OUT}/\partial T$, $R_{OUT}$ is the sensor’s output resistance, *K* is the ratio of the current mirrors (formed by *M2b* to *M2a* and *M1b* to *M1a*) within the temperature sensor, and ΔT is the temperature difference between transducers *Q1* and *Q2*, which is related to the power dissipated by the PA ($P_{PA}$) and with the thermal coupling resistance $R_{TH}$, which depends on the placement of devices within the IC and the materials that form the IC.

### 2.2. Experimental Characterization

**Figure 5:**
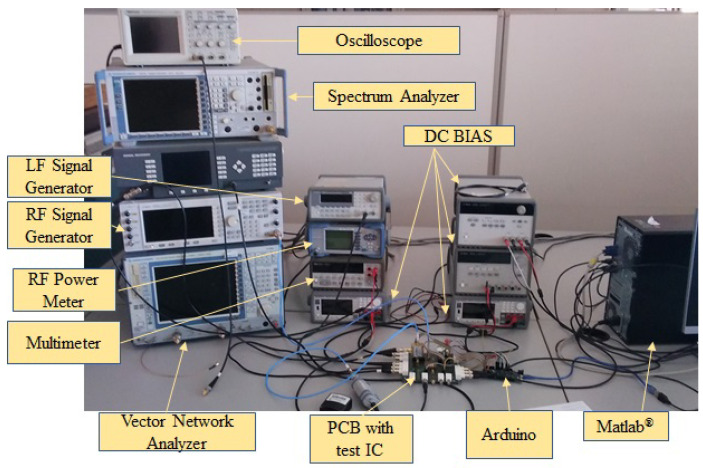
Laboratory setup and equipment used in the different experiments.

Figure 5 shows a photo of the experimental setup. The manufactured IC is mounted on a PCB board with connectors that allow access to the PA terminals (input, output, DC bias, $V_{DD}$) to bias the temperature sensor and to access its output voltage $V_{OUT}$. An Arduino microcontroller is used to generate the 10-bit digital number *N*, and a MATLAB^®^ code implements the control algorithm in Figure 1 and also commands all the equipment.

**Figure 6:**
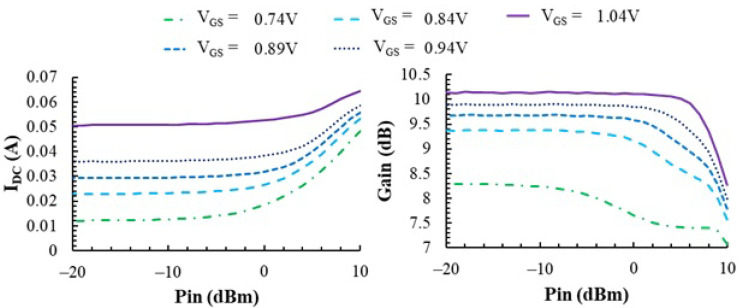
**Left**: Measurements of the DC (average) current in transistor *M_PA_*. **Right**: Power gain as a function of the input signal power *P_in_* at different operating points.

A DC voltage source is used to control the operating point of the amplifier (i.e., the $V_{GS}$ of $M_{PA}$ and $M_{ref}$) and thus its gain. Optimum input and output matching is produced at a frequency of 170 MHz; thus, all measurements reported in this paper are obtained at this frequency. The amplifier gain is obtained by measuring the power at the input and output ports with a power meter and is consistent with $s_{21}$ measurements with a 2-port network analyzer. Figure 6 shows the DC (average) current consumption $I_{DC}$ and gain of the PA at different bias points as a function of the input signal power $P_{in}$, measured in one of the IC samples. With small signals (low $P_{in}$), the gain can be increased by increasing $V_{GS}$ (i.e., $I_{DC}$) up to a maximum of 10.3 dB. At a nominal $V_{DD}$ = 3.3 V and $V_{GS}$ = 0.89 V, the gain of this sample is 9.3 dB. When increasing the signal power $P_{in}$, the PA eventually enters compression and gain is reduced. Note the compression point is higher when gate bias voltage increases, as then, the overdrive margin is increased and a larger signal is allowed before the NMOS enters the off state. Actually, this is the reason for the $I_{DC}$ increase with $P_{in}$ observed in Figure 6; i.e., when transistor $M_{PA}$ (see Figure 2) enters class-AB operation. This means that the $I_{DC}$ current in transistor $M_{PA}$ (and therefore, the power dissipated by the PA) actually depends both on the DC bias point and on the power of the input signal. This double dependence is relevant for the results presented in the following sections.

**Figure 7:**
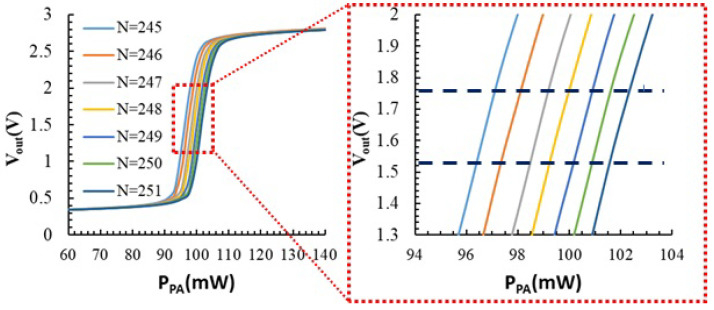
Sensor $V_{OUT}$ as a function of the power dissipated by the PA for 7 different values of *N*. Right: Zoom of the linear region of the function.

The evolution of the sensor output voltage as a function of the power dissipated by the PA is shown in Figure 7 for different values of *N*. The graphic only plots 7 of the 1024 possible values of *N*. For each value of *N*, the input linear range of the temperature sensor has an average value of 5.7 mW, and the average sensitivity is 275 V/W. At every increase of *N*, the sensor’s transfer function shifts to the right an average of 1.4 mW (magnitude of the horizontal axis).

**Figure 8:**
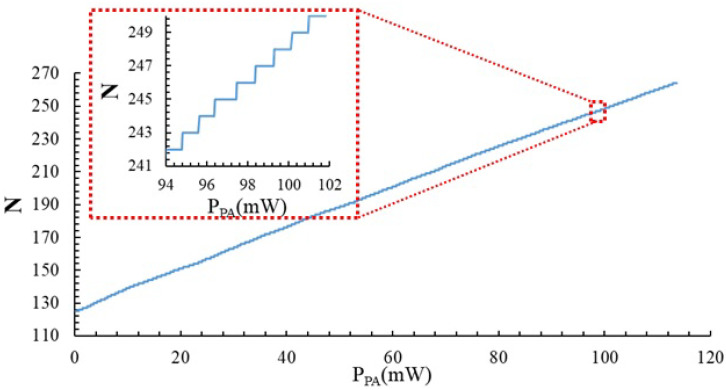
*N* values that set the sensor $V_{OUT}$ to $V_{DD}/2=$ 1.65 V (with a resolution of 10 mV) as a function of the power dissipated by the PA. Power changes due to the different values of $V_{GS}$.

The measurements in Figure 7 show how the thermal sensor can track variations of the power dissipated by the PA with high sensitivity, but the input dynamic range is low. To have a temperature sensor with wide dynamic range (i.e., being able to track variations of the power dissipated by the PA larger than 6 mW), we can use the digital value *N* as observable, taking advantage of the fact that for each value of power dissipated by the PA, there is an *N* value that grants that the sensor output voltage is close to $V_{DD}/2$ with a given resolution. Using this principle, we can use *N* to monitor the power dissipated by the PA, as illustrated in Figure 8 for one of the samples. In this example, the sensitivity of the sensor $\Delta N/\Delta P_{PA}$ is 1242 W^−1^. In the 11 samples measured, this sensitivity ranged from 817 W^−1^ to 1257 W^−1^. Using *N* temperature measurements, the sensor provides a wider input dynamic range, but the price is an increase in the uncertainty due to: (i) the quantization error, since the output *N* is now a discrete value; (ii) the non-uniform quantification step (as illustrated in the zoom in Figure 8), which results in non-linearity error; (iii) the resolution in the comparison of $V_{OUT}$ to $V_{DD}/2$, which is ±10 mV in our setup and (iv) the die-to-die variability in the sensitivity. We have estimated this overall uncertainty to be 4 mW, which is below the linear range of the sensor’s output voltage.

## 3. Strategies to Monitor Gain Variability with DC Temperature Measurements

### 3.1. Principle of the Technique

In order to monitor gain variations with temperature measurements, variations of gain must map into variations of the power dissipated by the PA. This can be obtained with two different strategies:

#### 3.1.1. Variation of the DC Operation Point

In a linear MOS amplifier, the voltage gain can be expressed as $|A_{v}|\approx g_{m}\cdot R_{L}$, with $R_{L}$ being the resistive load of the amplifier and $g_{m}$ the small-signal transconductance of the MOS transistor, which depends on the biasing current $I_{DC}$. Assuming long-channel and strong-inversion operation:

(3) $$g_{m}\approx \sqrt{2\cdot C_{OX}\cdot \mu_{n}\cdot \frac{W}{L}\cdot I_{DC}}$$

where $C_{OX}$ is the gate capacitance per unit area, *W* and *L* are, respectively, the MOS transistor width and length, and $\mu_{n}$ is the electron carrier mobility in the MOS channel. Therefore, by tracking the power variations produced by variations to $I_{DC}$, it is possible to track variations to the MOS transconductance and the gain, assuming the rest of the parameters remain constant.

#### 3.1.2. Variation of the Homodyne Dissipated Power

When an AC signal is applied to the PA input $\left(v_{in}=A\cdot cos\left(w_{s}\cdot t\right)\right)$, the DC power dissipated by the $M_{PA}$ transistor can be written as [26]:

(4) $$P_{PA}\approx V_{DD}\cdot I_{DC}-\frac{1}{2}\frac{A_{out}^{2}}{R_{L}}=V_{DD}\cdot I_{DC}-\frac{1}{2}\frac{A^{2}}{R_{L}}A_{v}^{2}$$

where $V_{DD}$ is 3.3 V, $I_{DC}$ is the DC bias current, $A_{out}$ is the amplitude of the AC signal at the output node (drain of $M_{PA}$), $R_{L}$ is the load resistance (50 Ω), and $A_{v}$ is the amplifier voltage gain. It is interesting to emphasize that the second term in Equation (4) cannot be measured by electrical means in the PA at DC since it contributes to the overall DC power dissipated by the amplifier but does not change either $V_{DD}$ or $I_{DC}$. From Equation (4), there is a relationship between the gain of the amplifier and the DC power dissipated by the PA when a signal is present.

### 3.2. Measurement Procedures to Track Gain Variation

After Figure 8, we can find a digital code $N_{1}$ that sets the sensor’s output voltage to $\frac{V_{DD}}{2}$ when the PA dissipates the power $P_{1}$. From this point on, we will use the term “*the N value measured when the dissipated power is P*”, assuming that for this value of *N* and this power dissipation *P*, the sensor $V_{OUT}$ is $\frac{V_{DD}}{2}$. However, dissipated power *P* can be the consequence of the DC current in the PA or the AC signal component; then, a suitable strategy must be defined to obtain a measurement indicative of the gain variation exclusively. Moreover, this strategy must be robust against the chip-to-chip variability in the PA and in the sensor circuit.

In this section, we define *Procedure1* and *Procedure2* to track the gain degradation along the lifetime of an amplifier using DC temperature measurements. They are based on the two strategies described above. The first procedure is based on measuring variations of the DC operating point of the MOS transistor within the PA, whereas the second is based on measuring variation of the homodyne dissipated power.

#### 3.2.1. Procedure1

We name $N_{off}$ the *N* value measured when the PA is not dissipating any power, whereas we name $N_{on}$ the *N* value measured when the PA is dissipating power due to its nominal biasing (*V_GS_* = 0.89 V, *V_DD_* = 3.3 V). In both cases, no AC signal is applied to the PA input. The difference $\Delta N=N_{on}-N_{off}$ is an indicator of the $I_{DC}$ current and, therefore, of the amplifier’s gain.

**Figure 9:**
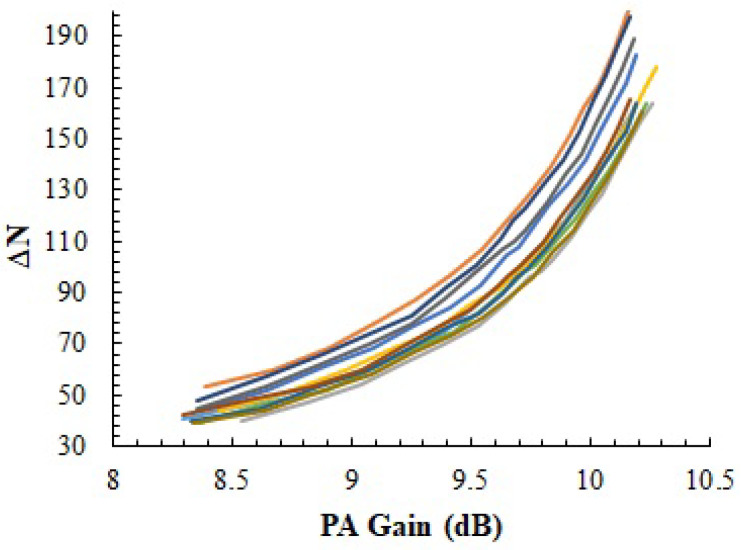
*N* as a function of the PA gain measured in 10 different IC samples. Each line in the figure corresponds to one sample.

**Figure 10:**
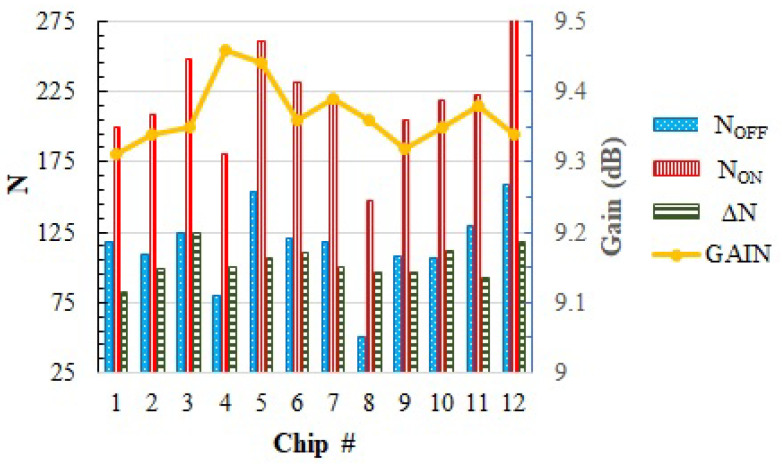
Variability of *Noff*, *Non*, ΔN and gain across different dies.

Figure 9 shows the results of these ΔN measurements as a function of the PA gain measured in 10 different IC samples. In order to change the gain of the amplifier, *V_GS_* is swept from 0.74 V to 1.04 V in 20 mV steps, providing a gain variation between 8.3 dB and 10.2 dB. ΔN measurements show the expected correlation with the gain: as the gain decreases (i.e., *I_DC_f* decreases), ΔN measurements decrease as well. However, an important die-to-die variability can be observed. For instance, when *V_GS_* = 0.89 V (nominal conditions, Figure 10), the gain of all amplifiers is in the range 9.31 dB to 9.45 dB, whereas the ΔN measurements go from 73 to 97. This important variability is associated with the high output resistance of the temperature sensor ($R_{OUT}$ in Equation (1)), and note how it impedes establishing a direct correlation between ΔN and the PA gain across different dies.

#### 3.2.2. Procedure2

**Figure 11:**
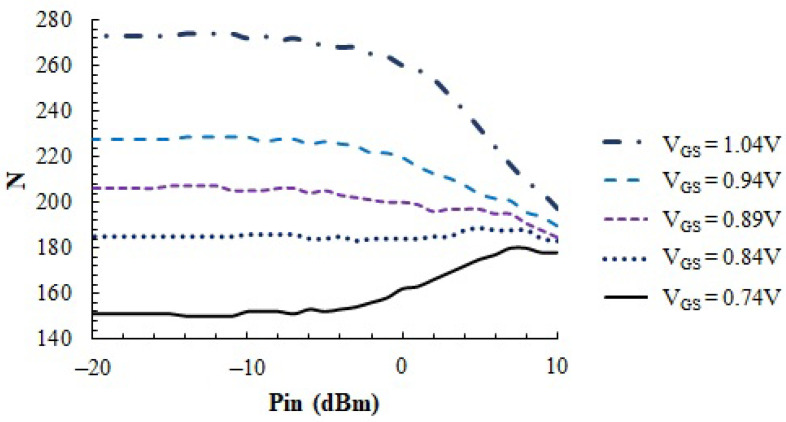
Evolution of the *N* as a function of the amplitude of the AC signal applied to the PA input. PA biased with five different *V_GS_* values.

When the PA is biased and an AC signal is applied to its input, according to Equation (4), a DC temperature measurement produces a value dependent on both the DC current and the signal response. In order to obtain temperature information only about the gain, ref. [26] proposed to perform two DC temperature measurements: one when the input AC signal has an amplitude $A_{1}$ and the second when the amplitude of the AC signal is $A_{2}$. If the $I_{DC}$ current remains constant, then the difference between both sensor readings provides information about the gain magnitude. However, in this PA, $I_{DC}$ depends on the input amplitude, as reported in Figure 6. To characterize the evolution of the two terms of Equation (4) as a function of the amplitude *A* for different gains, Figure 11 shows the *N* measurements as a function of the AC input signal amplitude for different PA biases (the ones reported in Figure 6).

Measurements show two different tendencies. On the one hand, for low $V_{GS}$ bias voltages, the *N* values increase as the AC amplitude of the signal applied to the PA input increases. This is the consequence of two facts: first, for these low $V_{GS}$ values, the gain of the amplifier is small; then the second term in Equation (4) is negligible, and the power dissipation depends mainly on $I_{DC}$. Second, as the AC signal amplitude is increased, the PA enters the class-AB operation regime; thus, $I_{DC}$ increases, as observed in Figure 6. On the contrary, for higher $V_{GS}$ bias voltages, gain increases and the amplifier remains in class-A operation for a larger range of input amplitudes. This causes the second term in Equation (4) to dominate, showing how, as reported in [26], the overall DC power dissipated by the PA decreases when the PA input amplitude increases.

As expected, *N* remains almost constant for $P_{in}$ levels lower than −10 dBm due to the resolution of about 1 mW reported in Section 2, but it shows a useful dependence on $P_{in}$ above this level, allowing us to track the power delivered to the load for input levels up to 10 dBm.

From the results in Figure 11, *Procedure2*: homodyne ΔN measurement ($P_{in_{1}},P_{in_{2}}$) is proposed:

1. Let us name as $N_{1}$ the *N* value measured when the AC input amplitude applied to the PA is $P_{in_{1}}$.
2. Let us name as $N_{2}$ the *N* value measured when the AC input amplitude applied to the PA is $P_{in_{2}}$.
3. The difference $\Delta N=N_{1}-N_{2}$ is an indication of the PA gain. If this difference changes, it implies that the PA gain has changed as well.

To use this technique, at least one of the $P_{in}$ values should be higher than −10 dBm.

**Figure 12:**
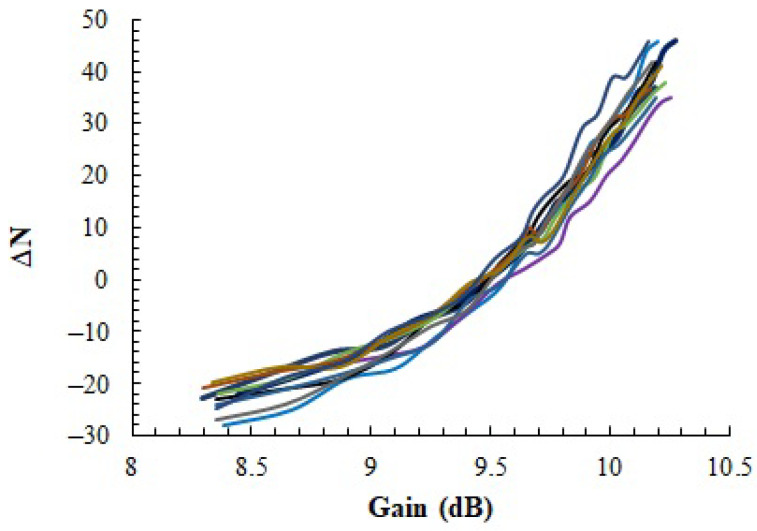
Results of homodyne *N* measurement (*Procedure2*): Dependency of the difference *N_1_*(@*P_in_1__* = −5 dBm)–*N_2_*(@*P_in_2__* = 5 dBm) as a function of the gain of the amplifier for 10 different samples. Each line in the figure corresponds to one sample.

The ΔN values measured after applying *Procedure2* (with $P_{in_{1}}$ = −5 dBm and $P_{in_{2}}$ = 5 dBm) are shown in Figure 12 as a function of the measured gain of the PA for 10 different IC samples. As in Figure 9, we biased the different samples with $V_{DD}$ = 3.3 V, while the gain of the PA was changed by sweeping $V_{GS}$ (from 0.74 V to 1.04 V with a 20 mV step). Although for some cases there are some noisy measurements, the tendency is very similar to that observed with *Procedure1*. The gain at which ΔN is zero is the one where the positive variation of the first term of Equation (4) cancels the negative variation of the second.

## 4. Aging Monitoring and Compensation

### 4.1. Experimental Measurements after Accelerated Aging

In order to test the above procedures under actual aging effects, the PA circuit in one of the IC samples was subjected to accelerated aging consisting of stressing the circuit with $V_{DD}$ = 5 V, $V_{GS}$ = 2.5 V, $P_{in}$ = 10 dBm (@freq = 170 MHz). The accelerated aging lasted a total of 60 min. To track the evolution of the PA figures of merit, we paused the stress at intermediate times *t* = 5, 10, 20, 30, 45 min from the start. During these pauses, we measured the intermediate degradation of the PA by performing characterization at the nominal bias (@$V_{DD}$ = 3.3 V, $V_{GS}$ = 0.89 V). After these characterization procedures at nominal conditions were performed, the accelerated aging conditions were resumed.

**Figure 13:**
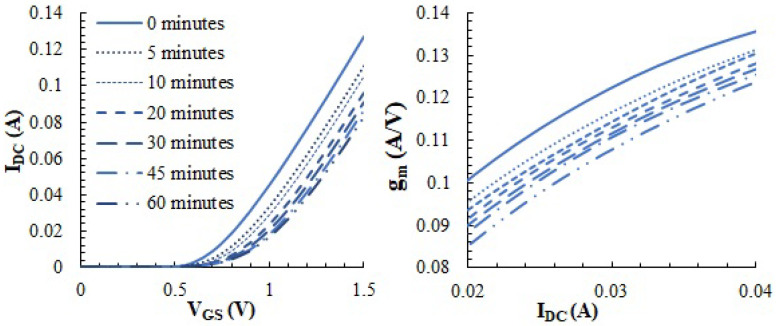
Evolution of the DC characteristics of the transistor *M_PA_* (**left**) and evolution of the MOS transconductance (**right**) as a function of the stress time (*V_DD_* = 3.3 V).

As discussed above, the voltage gain of a class-A amplifier is expected to be linearly related to the $g_{m}$ of transistor *M_PA_*, which, in turn, is related to the DC current at the bias point. Thus, we observed these three parameters to assess how they are affected by the aging process. Figure 13 (left) shows the evolution of the DC characteristics (@$V_{DD}$ = 3.3 V) of the PA transistor $M_{PA}$ extracted when the device was fresh (0 min), at the different pausing times within the stress procedure (at 5, 10, 20, 30, 45 min from the start) and at the end of the accelerated aging procedure (60 min). The plot shows the expected shift of the $I_{D}\left(V_{GS}\right)$ curves because of the degradation of the threshold voltage [3]. As a consequence, when biased at a constant $V_{GS}$ operating point, the DC current produced by the transistor suffers an important reduction. From this graph, we can also extract the MOS transconductance $g_{m}$ at any point in the curve, which is equal to $\partial I_{DC}\;/\;\partial V_{GS}$. This is plotted in Figure 13 (right) as a function of $I_{DC}$. The expected relationship between $g_{m}$ and $I_{DC}$ is shown, but it can also be observed how, even at a constant DC current, $g_{m}$ still degrades as a consequence of aging. According to Equation (3), this is indicative of a decrease in the carrier mobility $\mu_{n}$. While this aging effect on the mobility is well-known and reported in the literature [3,8,10], attention is often focused on the threshold voltage for degradation alone [9,30,31].

**Figure 14:**
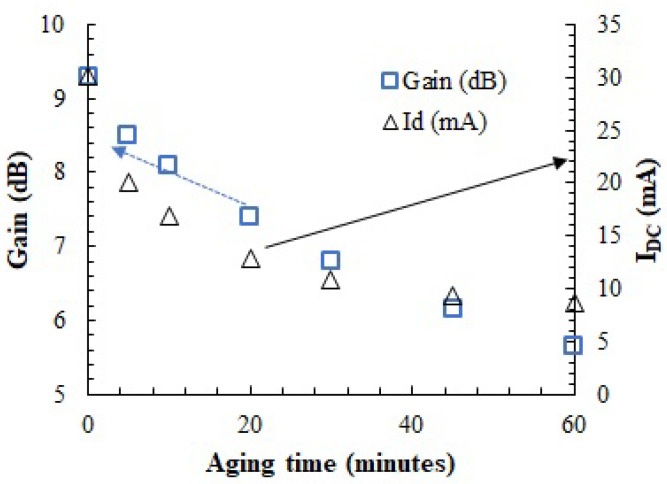
Evolution of the PA gain and *I_DC_* current at the nominal bias point (*V_DD_* = 3.3 V, *V_GS_* = 0.89 V).

Figure 14 plots the evolution of the DC drain current and gain measured on the PA as a function of the stress time when biased at nominal conditions (@$V_{DD}$ = 3.3 V; $V_{GS}$ = 0.89 V, freq = 170 MHz). After 1 h of stress, the PA gain decreased from 9.3 dB to 5.6 dB (57% degradation in linear units), whereas the drain current decreased from 30 mA to 8.6 mA (71% degradation).

### 4.2. Tracking Gain Degradation with Temperature Measurements

**Figure 15:**
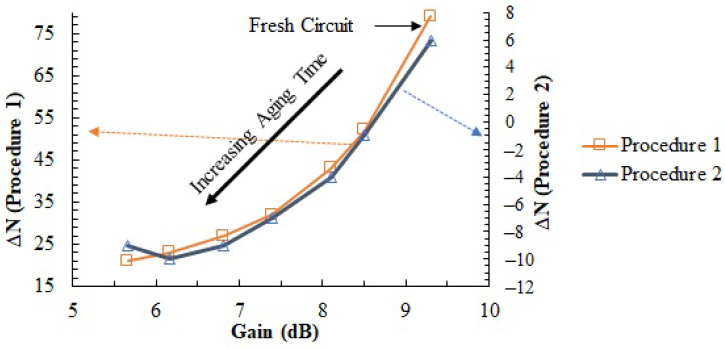
Evolution of the temperature measurements obtained in the two procedures proposed as a function of the measured PA gain degradation produced after stress was applied.

In order to assert if the degradation of the gain due to aging can be tracked with temperature measurements, we performed the two temperature measurement procedures described in Section 3 when the PA was fresh, in the pausing times within the aging and at the end of the stressing process. During all these temperature measurements, the PA was biased at the nominal conditions ($V_{DD}$ = 3.3 V; $V_{GS}$ = 0.89 V, freq = 170 MHz). The results obtained are shown in Figure 15, where the evolution of the sensor measurements as a function of the PA gain degradation is represented. The horizontal axis shows the different values of gain observed in the PA during the aging process. Both vertical axes show ΔN. The left vertical axis shows the values obtained with *Procedure1*, whereas the right vertical axis represents the values obtained with *Procedure2* ($P_{in_{1}}$ = −10 dBm, $P_{in_{2}}$ = 0 dBm). In both cases, the temperature measurements have the same monotonous correlation with the gain expected from the analysis in Section 3: as the gain degrades, the amplitude of the temperature difference measured is smaller. Note, this means that while direct ΔN cannot be directly correlated to the PA gain across different dies, it can be used to monitor the gain degradation within each PA.

The results demonstrate how the two measurement procedures produce an output reading indicative of the PA gain degradation due to aging, especially at the first moments of the aging process at which point the ratio between temperature and gain variations maximizes. In addition to this, we observe the tendency reported in Section 3 for *Procedure2*: When the PA is fresh, because the gain is high, the second term of Equation (4) dominates. When the gain of the amplifier has been strongly degraded due to aging, the first term of Equation (4) plays a major role in the evolution of the temperature measurements as a function of the gain. The threshold between both zones is the gain value where the ΔN measurement is zero.

### 4.3. Aging Compensation with Temperature Measurements

Figure 14 shows how the gain is degraded as the accelerated aging process goes on. If the aging procedure had been stopped at any of the different pausing times (5, 10, 20, 30, 45 or 60 min), we could have found a proper new $V_{GS}$ value that would restore the original gain. The question is, can we find this restoring $V_{GS}$ value, $V_{GS\_Restoring}$, only by observing temperature measurements and not by measuring the amplitude of the signal at the output of the PA?

To answer this question, we repeated the aging experiment in nine other IC samples and applied the following control algorithm using both gain-tracking procedures.

1. When the PA is fresh and biased with nominal conditions ($V_{GS}$ = 0.89 V, $V_{DD}$ = 3.3 V), we measure ΔN. We name this value the golden value, $\Delta N_{DC\_GOLDEN}$.
2. Accelerated aging is performed. As the circuit is aged, the measurement of ΔN produces a value smaller than the golden value.
3. We define initial values $V_{GS_{0}}$ = 0.89 V, $\Delta V_{GS_{0}}$ = 100 mV, i=j=0.
4. While $\Delta N_{i}$ is smaller than the golden value, we perform:
  (a) $V_{GS_{i+1}}=V_{GS_{i}}+\Delta V_{GS_{j}}$
  (b) i=i+1
  (c) $\Delta N_{i}$ = Temperature measurement$\left(V_{GS_{i}}\right)$
5. If $\Delta N_{i}$ is equal to $\Delta N_{DC\_GOLDEN}$, then $V_{GS\_Restoring}=V_{GS_{i}}$. Otherwise, it means that $\Delta N_{i}$ is larger than $\Delta N_{DC\_GOLDEN}$. Then, we refine our search:
  (a) $\Delta V_{GS_{j+1}}=\frac{\Delta V_{GS_{j}}}{2}$
  (b) j=j+1
6. While $\Delta N_{i}$ is larger than the golden value, we perform
  (a) $V_{GS_{i+1}}=V_{GS_{i}}-\Delta V_{GS_{j}}$
  (b) i=i+1
  (c) $\Delta N_{i}$ = Temperature measurement$\left(V_{GS_{i}}\right)$
7. If $\Delta N_{i}$ is equal to $\Delta N_{DC\_GOLDEN}$, then $V_{GS\_Restoring}=V_{GS_{i}}$. Otherwise, it means that $\Delta N_{i}$ is smaller than $\Delta N_{DC\_GOLDEN}$. Then, we again refine our search:
  (a) $\Delta V_{GS_{j+1}}=\frac{\Delta V_{GS_{j}}}{2}$
  (b) j=j+1
8. Go back to Point 4.

**Figure 16:**
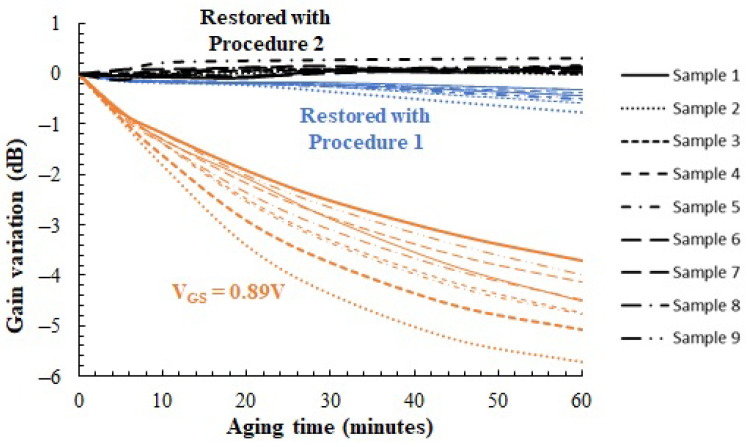
Evolution of the gain for 9 samples vs. aging time in three situations. (i) $V_{GS}$ is 0.89 V. (ii) $V_{GS\_Restoring}$ when *Procedure1* is used. (iii) $V_{GS\_Restoring}$ when *Procedure2* is used. Pattern line identifies the sample number. Color identifies the situation.

Figure 16 shows the evolution of the gain measured on the nine different samples as a function of the aging time when the PA is biased: (i) with constant $V_{GS}$ at 0.89 V; (ii) with the $V_{GS\_Restoring}$ obtained using *Procedure1*; and (iii) with the $V_{GS\_Restoring}$ obtained using *Procedure2*. Data are normalized to the gain of each PA when fresh (the fresh gain of the samples is between 9.31 and 9.45 dB, Figure 10, due to die variability). It can be observed how when no aging compensation procedures are used, the gain degradation observed in the different samples ranges from 3.7 dB to 5.7 dB after 1 h of stress (from 57% to 73% in linear units). However, results show how *Procedure1* and *Procedure2* are able to ensure roughly constant gain despite the aging suffered by the PA circuit. After *Procedure1*, the differences in the fresh gain after 1 h of stress are only between 0.32 and 0.76 dB (from 7% to 16% in linear units), with the restored gain being a slightly underestimated version of the fresh one. Actually, the error in the nominal gain tends to increase with the aging time. This is because the target of *Procedure1* is to ensure that the aged PA has the same power dissipation due to DC bias as the fresh one, i.e., it only restores the fresh $I_{DC}$ value. As shown previously in Figure 13, restoring the $I_{DC}$ alone does not restore the small-signal transconductance $g_{m}$ because of its additional dependence on the carrier mobility $\mu_{n}$ (Equation (3)), which degrades as well. On the contrary, *Procedure2* provides much better gain healing, as the homodyne dissipated power (Equation (4)) does contain information about the gain. Using *Procedure2*, the self-healing algorithm is able to set the gain with an error below 0.3 dB along all the stress times (less than 6% deviation), which is basically limited by the sources of uncertainty in the temperature sensor described in Section 2.

While *Procedure1* compensates the gain with some error, this is small enough to be commonly accepted in the context of RF PAs and shows the advantage of a simpler implementation, as the procedure does not require any AC input signal. *Procedure2* guarantees accurate correction of the gain at the cost of a somewhat more complex procedure. In any event, both self-healing procedures have been demonstrated to recover the amplifier gain automatically with a different accuracy and complexity trade-off and thus can be used to keep the amplifier’s gain constant along time despite aging.

## 5. Conclusions

In this paper, we have presented an experimental demonstration of how temperature measurements can be used to track gain variation in an amplifier due to circuit aging. Furthermore, we have shown how these temperature observations can be used to control an adaptive biasing scheme that preserves constant amplification despite aging circuit degradation. For that purpose, we have proposed and tested two different measurement procedures, both based on measuring the DC output of a temperature sensor. In the example of this paper, the frequency of operation of the amplifier is 170 MHz, but the procedures have no limit to the frequency of operation of the circuit under test thanks to the inherent conversion from high-frequency voltage and current signals to DC power dissipation given by the Joule Effect.

The first procedure has the distinctive advantage that it does not need any AC stimulus of the monitored circuit. Moreover, in class-A circuits such as the one used as an example in this paper, it is the procedure that requires a temperature sensor with the smallest sensitivity, as the measured temperature increases are bigger. However, it has proven to be slightly less effective in retrieving the gain of the fresh circuit due to the fact that it reestablishes the original DC operating point but does not consider the small-signal response of the circuit. On the contrary, the second procedure proposed, based on homodyne temperature measurements, is sensitive to the AC circuit response, and thus, it is able to preserve constant gain with deviation less than 0.3 dB according to our measurements. This procedure, however, requires a temperature sensor with higher sensitivity.

The measurement procedure to use depends on the characteristics of the temperature sensor available, but the two alternatives offer an interesting trade-off between simplicity and accuracy. The sensor used in this paper has been used as a proof-of-concept: thanks to the fact that it provides high sensitivity and a wide dynamic range, it has allowed us to test both procedures.

To the best of the authors’ knowledge, this is the first work that has performed accelerated aging on CMOS PA circuits, measured the important gain degradation, and then used a noninvasive sensor and automatic self-healing to demonstrate how the gain can be recovered and kept constant along time despite the circuit aging. The results of this work open a new scenario to use temperature sensors that are already placed in integrated circuits for purposes of reliability monitoring and healing.

## 6. Patents

The work reported in this manuscript is present in Spanish Patent P2022311016. 

## Data Availability

Not applicable.

## Abbreviations

The following abbreviations are used in this manuscript:

CMOS	Complementary Metal-Oxide Semiconductor
DC	Direct Current
IC	Integrated Circuit
PVT	Process Voltage Temperature
HCI	Hot Carrier Injection
BTI	Bias Temperature Instability
TDDB	Time-Dependent Dielectric Breakdown
EM	Electro-Migration
PA	Power Amplifier
IR	Infra Red
RF	Radio Frequency
MOS	Metal-Oxide Semiconductor
CUT	Circuit Under Test
OTA	Operational Transconductance Amplifier
AC	Alternating Current
